# Investigation of the Cytotoxicity of Cu(II), Au(III), and Pd(II) Complexes with 2,4-Dithiouracil and 6-Propyl-2-thiouracil Derivatives

**DOI:** 10.3390/biotech14030053

**Published:** 2025-07-01

**Authors:** Petya Marinova, Denica Blazheva, Aleksandar Slavchev, Petia Genova-Kalou

**Affiliations:** 1 Department of General and Inorganic Chemistry with Methodology of Chemistry Education, Faculty of Chemistry, University of Plovdiv, “Tzar Assen” str. 24, 4000 Plovdiv, Bulgaria; 2 Department of Microbiology and Biotechnology, University of Food Technologies, 26 Maritza Blvd., 4002 Plovdiv, Bulgaria; d_blazheva@uft-plovdiv.bg (D.B.); a_slavchev@uft-plovdiv.bg (A.S.); 3 National Reference Laboratory “Rickettsia and Cell Cultures”, Department of Virology, National Centre of Infectious and Parasitic Diseases, 1504 Sofia, Bulgaria; petia.d.genova@abv.bg

**Keywords:** 6-propyl-2-thiouracil, 2,4-dithiouracil, copper(II) complexes, palladium(II) complexes, gold(III) complexes, cytotoxic properties

## Abstract

This study investigates the cytotoxic properties of metal complexes incorporating thio-uracil derivatives, specifically 2,4-dithiouracil and 6-propyl-2-thiouracil. The research focuses on the cytotoxic effects of Cu(II) and Pd(II) complexes with 6-propyl-2-thiouracil, as well as mixed-ligand transition metal Cu(II) and Au(III) complexes of 2,4-dithiouracil with 2-thiouracil and uracil. Cytotoxic activity was assessed against human cervical carcinoma cells (HeLa) and normal kidney cells from the African green monkey. The results demonstrated that incorporating Cu(II) and Au(III) into the compound structures significantly enhanced their cytotoxic effects. Notably, all tested complexes exhibited a stronger inhibitory effect on cancer cell proliferation compared to normal cells, with the palladium(II) complex of 6-propyl-2-thiouracil showing the lowest CD_50_ value against the tumor cell line (0.00064 mM), which were 149 times lower than that of the ligand (0.0955 mM). These findings suggest that thio-uracil-based metal complexes, particularly those containing palladium (II) and gold(III), hold significant potential for further development as anticancer agents.

## 1. Introduction

**Scheme 1:**
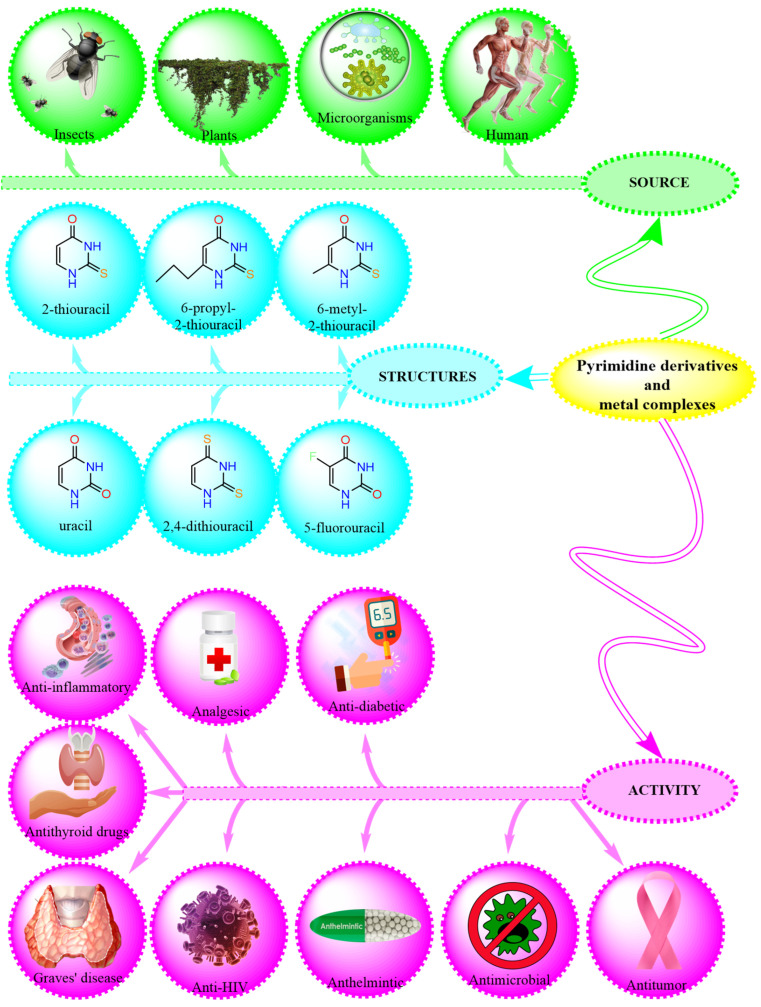
Different applications and structures of pyrimidine derivatives and their metal complexes.

Thionamides, a category of relatively straightforward molecules, function as antithyroid medications, incorporating a sulfhydryl group and a thiourea unit within a heterocyclic structure [1,2,3,4]. The interest in platinum and palladium compounds arises from their notable cytostatic properties [5]. In Scheme 1, the different applications of bioactive pyrimidines, such as uracil, 2,4-dithiouracil, 6-propyl-2-thiouracil, and other pyrimidines and their metal complexes, is given [6,7,8,9,10]. These compounds are modifications of the pyrimidine ring, which are often used in medicinal chemistry and drug design.

Recently, cis-dihalogenated complexes of Pd(II) and Pt(II) were investigated in combination with 6-tert-butyl-2-thiouracil [11]. Vetter et al. described the synthesis, characterization, and in vitro study of the cytotoxic potential of platinum(IV) complexes with thiouracil-based ligands [12]. Ru(II)-centered complexes incorporating antimicrobal 2-thiouracil derivatives—specifically 6-methyl-2-thiouracil (6m2TU) and 2-thiouracil (2TU)—were tested against HepG2 (human hepatocellular carcinoma), K-562 (human chronic myeloid leukemia), HL-60 (human promyelocytic leukemia), and B16-F10 (mouse melanoma) cancer cells, as well as non-cancerous cells (PBMCs) (human peripheral blood mononuclear cells activated with concanavalin A—human lymphoblasts) [13]. The biological evaluation indicated that a complex with 6-methyl-2-thiouracil demonstrated greater potential than a complex with 2-thiouracil. Ultimately, this investigation suggests that complexes with 2-thiouracil and 6-methyl-2-thiouracil induce apoptosis, significantly increasing the proportion of apoptotic HL-60 cells by disrupting the cell cycle and decreasing the presence of cells in the G_1_/G_0_, G_2_/M, and S phases [13]. Correa et al. presented the chemical and cytotoxic investigations of four novel ruthenium(II) complexes incorporating uracil derivatives [4]. In vitro studies tested their activity against HL-60, HepG2 (human liver cancer), B16-F10, and K562 cells, along with non-cancerous PBMCs [14]. Bomfim et al. synthesized Ru(II) complexes incorporating 6-methyl-2-thiouracil, demonstrating their potential as innovative antileukemic drug candidates [15]. Oladipo and Isola carried out a comprehensive review of uracil’s coordination chemistry and the practical uses of several of its complexes [16]. 5-substituted uracils are recognized as key structural components in various therapeutics [17]. Prachayasittikul et al. synthesized novel mixed-ligand transition metal (Cu, Ni, and Mn) complexes of 5-iodouracil (5Iu) with 8-hydroxyquinoline (8HQ) and 5-nitrouracil (5Nu) with 8HQ. These metal complexes showed notable cytotoxic effect against HuCCA-1, A-549, HepG2, and MOLT-3 cell lines [7]. Al-Halbosy et al. presented the cytotoxic effect of the N-Phenylmorpholine-4-carbothioamide (HPMCT) and PdCl_2_(HPMCT)_2_] and [PtCl_2_(HPMCT)_2_] complexes. The compounds were assessed on breast cancer cell lines (MCF-7), and a complex with a palladium ion revealed the most promising activity with an IC_50_ value 12.72 ± 0.4 μM [18]. Copper complexes exhibit cytotoxic activity through mechanisms distinct from those employed by cisplatin, which is a platinum-based chemotherapeutic agent currently in clinical use [19]. The biological efficacy of these copper compounds varies significantly depending on the nature of the coordinated ligands. Investigations into their cytotoxic potential are often grounded in the hypothesis that endogenous copper may exert lower toxicity on normal cells compared to malignant ones [20]. The involvement of copper in angiogenesis remains a matter of scientific debate, and the broader role of transition metals in this process continues to be actively explored [20]. Notably, copper complexes have been shown to mimic the activity of superoxide dismutase (SOD) [21], which is a key antioxidant enzyme that catalyzes the dismutation of superoxide radicals into less reactive molecular species.

Recently, a complex with the general formulae [Cu(C_20_H_22_NO_3_)_2_]_·_H_2_O was obtained and its cytotoxic properties were assessed [22]. The in vitro cytotoxic investigation was conducted using an MTT assay, and the result revealed that a metal complex exhibited enhanced cytotoxicity, high selectivity, and dose-dependent cytotoxicity [22]. Zou et al. described gold complexes in detail in a recent review [23]. Many Au(III) complexes were synthesized, and the anticancer effect was assisted against different cancer cell lines. In most cases, the ligands contained a donor atom, either Cl, Br, S, or P. da Silva Maia et al. have also synthesized gold(III) complexes, and their cytotoxic effects were studied [24]. Most of the cited gold(III) complexes had a profound effect on cisplatin-resistant cell lines. The cytotoxic properties of a complex with the general formulae [(η-C_5_H_5_)_2_Ti{OC(O)CH_2_PPh_2_AuCl}_2_] was investigated in vitro against prostate and renal cell lines as potential chemotherapeutics drugs [25]. The result showed that the complex acts synergistically because the resulting cytotoxic effect is more pronounced when compared to [{HOC(O)RPPh_2_}AuCl] (R = −CH_2_−6, −4-C_6_H_4_−7) in renal cancer cell lines [25]. Metal complexes tend to associate with specific residues, many of which play key roles in the enzyme’s catalytic function. This interaction can disrupt cellular activities, ultimately leading to programmed cell death (apoptosis) [26]. Rana et al. investigated a gold(III) complex known to bind to TrxR [26]. In this enzyme, the catalytic residues were positioned between two subunits, each contributing to the gold(III) complex attachment. To date, numerous metal complexes have been developed utilizing uracil and thiouracil derivatives, incorporating various metals such as Cu, Fe, Co, Ni, Zn, Mn, Cd, and V [27,28,29], along with Pd, Pt, and Au, with analyses conducted on their composition and structural characteristics [30]. Novel thiolate gold(I) complexes, incorporating P(NMe_2_)_3_ (HMPT) as a phosphine ligand, were successfully synthesized [31], with two of these gold(I) thiolate complexes exhibiting potential as promising chemotherapy agents. Additionally, the cytotoxic effect of various metal complexes derived from thiouracil have been investigated against different cancer cell lines [31,32,33,34,35]. Recently, Marinova et al. successfully synthesized copper(II), palladium(II), and gold(III) complexes with 2-thiouracil [36], along with novel palladium(II) and copper(II) complexes containing 6-propyl-2-thiouracil and 6-methyl-2-thiouracil [37]. Additionally, Au(III) and Cu(II) complexes incorporating 2,4-dithiouracil (2,4-DTu) were developed [38], as well as an Au(III) complex with 6-methyl-2-thioxo-2,3-dihydropyrimidin-4(1*H*)-one [39]. Furthermore, the biological effect and synthesis of several metal complexes derived from 2-thiouracil and its derivatives were explored [40]. Adhikari et al. carried out a comprehensive review of Pt, Ru, Au, Cu, Ir, and Os coordination compounds and its anti-cancer activities [41]. Numerous Au(I) and Au(III) complexes have been obtained and investigated as possible antitumor therapies in recent years [42,43,44,45].

**Figure 1:**
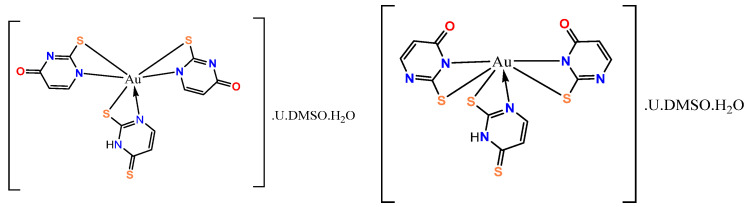
The probable structure of the gold complex with 2,4-dithiouracil, 2-Tu, and U [38].

**Figure 2:**
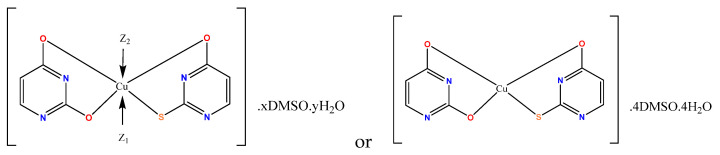
A proposed structural arrangement and the corresponding coordination binding sites in the copper complex. If Z_1_ = H_2_O or DMSO-h_6_ then Z_2_ = H_2_O or DMSO-h_6_ [38].

**Figure 3:**
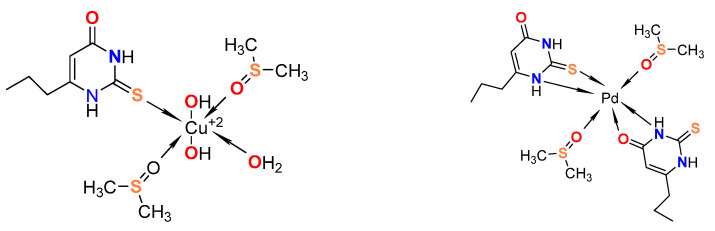
The illustration of the suggested coordination binding sites for 6-propyl-2-thiouracil with copper and palladium [37].

This article describes the cytotoxic properties of metal compounds with 2,4-dithiouracil and 6-propyl-2-thiouracil. In this study, we provide details regarding the cytotoxic effect of Pd(II) and Cu(II) complexes with 6-propyl-2-thiouracil and Au(III) and Cu(II) complexes with 2,4-dithiouracil. The possible structures of the gold and copper complexes with 2,4-dithiouracil are shown in Figure 1 and Figure 2, respectively, and Cu(II) and Pd(II) complexes with 6-propyl-2-thiouracil are shown in Figure 3. All complexes that are the subject of this study have been previously published by us in two articles [37,38]. In our previous study, it was proposed that 2,4-dithiouracil might undergo desulfurization due to the action of the NaOH employed during the synthesis of the gold complex. This process could lead to the substitution of one or both sulfur atoms with oxygen, thereby yielding 2-thiouracil and uracil as potential products within the reaction mixture [38]. The structures of the present complexes are discussed based on melting point analysis, microwave plasma atomic emission spectrometry (MP-AES) for Cu and Pd, inductively coupled plasma optical emission spectrometry (ICP-OES) for S (complexes with 2,4-dithiouracil), solution and solid-state NMR, attenuated total reflection (ATR), and Raman spectroscopy [37,38].

## 2. Materials and Methods

### 2.1. Cytotoxicity Assay

To assess the in vitro biocompatibility of the tested compounds, a series of cell viability assays were conducted on human cervical carcinoma cell lines (HeLa), as well as on normal Vero cells (kidney cells from the African green monkey). All cell lines were obtained from the German Collection of Microorganisms and Cell Cultures (DSMZ GmbH, Braunschweig, Germany). The cell cultures were maintained in DMEM (Dulbecco’s Modified Eagle Medium) (Gibco Thermofisher Scientific, 168 Third Avenue, Waltham, MA, USA, 02451) growth medium, supplemented with 10% fetal bovine serum (FBS, Sigma-Aldrich, Darmstadt, Germany) and 5% L-glutamine (Sigma-Aldrich, Darmstadt, Germany) and incubated under controlled conditions of 37 °C in a humidified atmosphere with 5% CO_2_.

### 2.2. Cell Viability Assessment

The experimental approach included a series of cytotoxicity assays to determine the extent of cell proliferation inhibition by the synthesized complexes and their free ligands. Cell viability was quantified using a standard MTT-based colorimetric assay. Exponentially growing cells were harvested and seeded (100 μL per well) into 96-well plates at an appropriate density—3 × 10^5^ for HeLa and Vero cells. The cells were exposed to various concentrations of the tested compounds, ranging from 0.0001 to 10 mg/mL, and incubated for 72 h.

Following the incubation period, a filter-sterilized MTT substrate solution (5 mg/mL in PBS (Sigma-Aldrich, Darmstadt, Germany) was introduced into each well. A subsequent incubation of 1–4 h facilitated the formation of insoluble purple formazan crystals, which were then dissolved in an isopropanol solution containing 5% formic acid. Absorbance measurements were taken at 550 nm using a microplate reader (Labexim LMR-1). The absorbance values were corrected against MTT and isopropanol controls and normalized to the mean value of the untreated control (100% cell viability). Semi-logarithmic dose–response curves were generated, and the cytotoxic dose CD_50_ (causing a 50% reduction in cell viability) for each tested compound against the respective cell lines was determined. Statistical significance was established at *p* ≤ 0.05.

## 3. Results and Discussion

**Figure 4:**
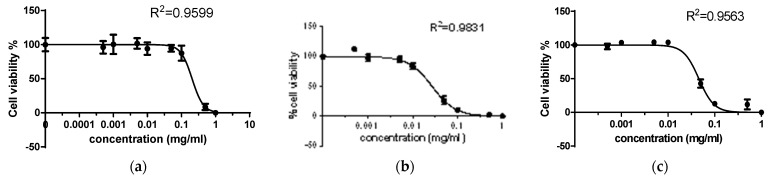
Concentration–response curves of (**a**) 2,4-dithiouracil and its (**b**) Cu(II) and (**c**) Au(III) complex against the Vero cell line (kidney cells from African green monkey).

**Figure 5:**
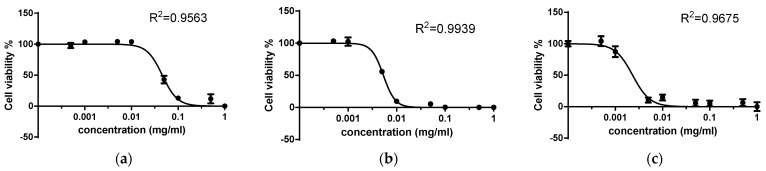
Concentration–response curves of (**a**) 2,4-dithiouracil and its (**b**) Cu(II) and (**c**) Au(III) complex against the HeLa cell line (human cervical carcinoma).The CD_50_ values of the 2,4-dithiouracil ligand and its metal complexes were determined through MTT testing and are presented in Table 1. The hierarchal order of cytotoxicity against the normal cell line revealed that the addition of Cu(II) increased the cytotoxic activity of the ligand and the addition of Au(III) to the complex led to an even lower CD_50_. The increase in activity was 37-hold for the Au(III) complex and 36-fold for the Cu(II) complex in comparison with the ligand. The tumor cell line was also less sensitive to 2,4-dithiouracil in comparison with its complexes, with the Au(III) complex being the stronger cytotoxic agent. The cytotoxic effect against the tumor line of the Cu(II) complex was 41 times stronger than that of the ligand, and the addition of Au(III) to the complex resulted in almost 162 times higher activity. Other authors also reported an improvement of cytotoxicity after the addition of copper and gold ions to ligands [20,23,41]. The comparison between the normal and tumor line sensitivity towards 2,4-dithiouracil and its metal complexes show much higher cytotoxicity against human cervical carcinoma cells, which is a good prerequisite for possible chemotherapy applications.

Two thiouracil derivatives and their complexes with Au(III), Pd(II), and Cu(II) were tested for cytotoxicity against two cell lines—a normal cell line of kidney cells from African green monkey and a tumor cell line of human cervical carcinoma. The concentration–response curves for 2,4-dithiouracil and its metal complexes against the normal cell line are presented on Figure 4. The results show that the tested compounds reduced the proliferation of the treated cells in a concentration-dependent manner, but the addition of Cu(II) and Au(III) improved cell viability at the higher tested concentrations. A similar tendency was observed in the treatment of the tumor cell line (human cervical carcinoma) (Figure 5) with the same compounds.

**Figure 6:**
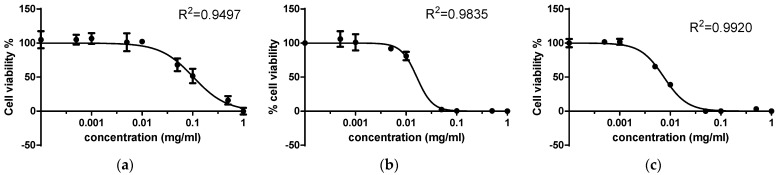
Concentration–response curves of (**a**) 6-propyl-2-thiouracil and its (**b**) Cu (II) and (**c**) Pd (II) complex against the Vero cell line (kidney cells from African green monkey).

**Figure 7:**
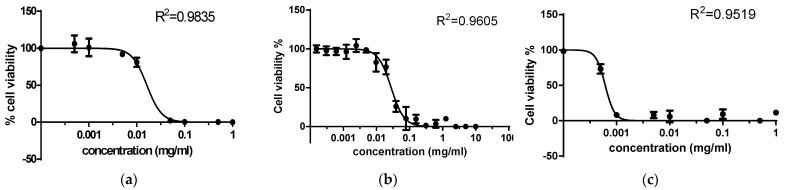
Concentration–response curves of (**a**) 6-propyl-2-thiouracil and its (**b**) Cu(II) and (**c**) Pd(II) complex against the HeLa cell line (human cervical carcinoma).

Similar results were obtained in the investigation of the cytotoxic effect of 6-propyl-2-thiouracil and its complexes with copper(II) and palladium(II). The concentration–response curves depicted in Figure 6 reveal that the cell viability of the normal cell line is also concentration-dependent and the metal complexes are cytotoxic in lower concentration in comparison with the ligand. In the experiments with tumor cells and the same compounds, the cell viability was affected by even lower test concentrations (Figure 7).

The determined CD_50_ values of 6-propyl-2-thiouracil and its complexes were lower than those of 2,4-DTu and its Pd(II) and Cu(II) compounds. The highest cytotoxic activity against the normal cell line was detected for Pd(II) complex, followed by its Cu(II) and 6-Pro-2Tu (Table 2). The addition of Cu(II) to the ligand increased its activity 17 times, while the inclusion of Pd(II) contributed to an almost 78 times increase. Similarly, the most potent compound preventing the proliferation of the tumor cells was the Pd(II) complex of 6-propyl-2-thiouracil, followed by the Cu(II) complex and the ligand. The Cu(II) complex exhibited a 9-fold higher cytotoxic activity against the tumor cell line, and the Pd(II) complex exhibited a more than 149-fold higher cytotoxic activity. This second set of compounds also showed a better effect on tumor cells in comparison with the normal cells.

**Scheme 2:**
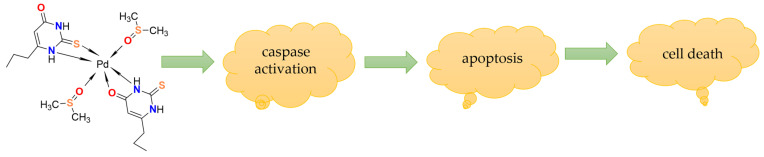
Proposed mechanism of the cytotoxic action of the Pd(II) complex.

The cytotoxic activity against the normal cell line of kidney cells from African green monkey of the 6-propyl-2-thiouracil was two times stronger than that of the 2,4-dithiouracil and three times against the HeLa tumor cell line. When the copper complex of 6-propyl-2-thiouracil is compared to that with 2,4-DTu, it can be noted that the cytotoxic effect of the Cu(II) complex with 6-Pro-2-Tu against the normal cell line and the human cervical cell line was almost the same compared to the Cu(II) complex with 2,4-DTu. It should be noted that the Pd(II) complex with 6-propyl-2-thiouracil exhibited a 149-fold higher cytotoxic activity against the tumor cell line HeLa. The proposed mechanism of the cytotoxic action of the Pd(II) complex is given in Scheme 2.

Cisplatin is a widely used chemotherapeutic agent, and its cytotoxic effects on HeLa cell lines have been extensively studied [46,47,48,49,50,51]. On average, a concentration of 0.003 mg/mL is reported to lead to a significant loss of cell viability in HeLa cell lines. Becit et al. reported that for complete inhibition of the viability of a human cervical carcinoma cell line, a concentration of cisplatin higher than 1.5 mg/mL was needed [52]. Another study reported that 0.15 mg/mL of cisplatin caused about 10% reduction in cell viability in the same cell line after 24 h [53]. In comparison, the compound included in this study completely inhibited the proliferation of the tumor line at concentrations between 0.001 and 0.1 mg/mL. Another platinum-based chemotherapeutic agent that exhibits cytotoxic effects on HeLa cells is carboplatin. Various potency levels have been reported across different studies. Aborehab and Osama [54] investigated the effect of combining gallic acid with carboplatin on HeLa cells, employing concentrations of carboplatin ranging from 0.00781 mg/mL to 1 mg/mL. The application of 1 mg/mL yielded complete inhibition of cell viability, which is a significantly higher concentration than the ones reported in this study.

## 4. Conclusions

The conducted study on 2-thiouracil derivatives and their metal complexes showed that the inclusion of Cu(II), Pd(II), and Au(III) in the compounds structure significantly improved their cytotoxicity. All tested compounds exhibited a higher capability to reduce the proliferation of human cervical carcinoma cells in comparison with normal kidney cells from African green monkey. The cytotoxic activity against the tumor cell line of human cervical carcinoma of the Cu(II) complex was 41 times stronger than that of the 2,4-dithiouracil, and the addition of Au(III) to the complex resulted in an almost 162 times higher effect. The Cu(II) complex with 6-propyl-2-thiouracil exhibited a 9-fold higher cytotoxic activity against the tumor cell line HeLa, and the Pd(II) complex was more than 149-fold. The lowest CD_50_ detected was for the complex of 6-propyl-2-thiouracil with Pd(II) against the tumor cell line—0.00064 mM. These findings suggest that the coordination of metal ions, especially Cu(II), Au(III), and Pd(II) to 2-thiouracil derivatives, plays a significant role in the improvement of their cytotoxicity towards tumor cells while maintaining lower toxicity towards normal cells. This makes them promising candidates for further investigation as anticancer agents.

## Figures and Tables

**Table 1:** Hierarchical order of 2,4-dithiouracil and its complexes according to their cytotoxicity against the Vero cell line (kidney cells from African green monkey).

Compound	Cytotoxic Concentration (CD_50_), mM	
	Normal Cell Line	Tumor Cell Line
	Vero Cell Line (Kidney Cells from African Green Monkey)	HeLa Cell Line (Human Cervical Carcinoma)
2,4-dithiouracil	1.42075	0.31674
Cu(II) complex	0.0396	0.0078
Au(III) complex	0.0385	0.00196
Order	Au(III) complex > Cu(II) complex > 2,4-DTu	Au(III) complex > Cu(II) complex > 2,4-DTu

**Table 2:** Hierarchical order of 6-Pro-2Tu and its complexes according to their cytotoxicity against the Vero cell line (kidney cells from African green monkey).

Compound	Cytotoxic Concentration (CD_50_), mM	
	Normal Cell Line	Tumor Cell line
	Vero Cell Line (Kidney Cells from African Green Monkey)	HeLa Cell Line (Human Cervical Carcinoma)
6-propyl-2-thiouracil	0.60683	0.0955
Cu(II) complex	0.0368	0.0113
Pd(II) complex	0.00779	0.00064
Order	Pd(II) complex > Cu(II) complex > 6-Pro-2Tu	Pd(II) complex > Cu(II) complex > 6-Pro-2Tu

## Data Availability

The original contributions presented in this study are included in this article. Further inquiries can be directed to the corresponding author.
